# MicroRNA Biomarkers of High-Grade Cervical Intraepithelial Neoplasia in Liquid Biopsy

**DOI:** 10.1155/2021/6650966

**Published:** 2021-04-13

**Authors:** Rhafaela L. Causin, Luciane S. da Silva, Adriane F. Evangelista, Letícia F. Leal, Karen C. B. Souza, Danielle Pessôa-Pereira, Graziela M. Matsushita, Rui M. Reis, José H. T. G. Fregnani, Márcia M. C. Marques

**Affiliations:** ^1^Molecular Oncology Research Center, Barretos Cancer Hospital, Barretos, SP 14784-400, Brazil; ^2^Department of Pathology, Barretos Cancer Hospital, Barretos, SP 14784-400, Brazil; ^3^Life and Health Sciences Research Institute (ICVS), Medical School, University of Minho, Braga, Portugal. ICVS/3B's-PT Government Associate Laboratory, Braga, Guimarães, Portugal; ^4^A. C. Camargo Cancer Center, São Paulo, SP 01509-010, Brazil; ^5^Barretos School of Health Sciences, Dr. Paulo Prata-FACISB, Barretos, SP 14785-002, Brazil

## Abstract

New prevention strategies are needed to detect cervical intraepithelial neoplasia (CIN). The microRNA expression analysis has already been reported as molecular biomarkers in the early detection of cervical cancer (CC) through minimally invasive samples, such as liquid biopsy, obtained through collection using liquid-based cytology (LBC). In this study, we aimed to identify molecular signatures of microRNAs in cervical precursor lesions from LBC cervical and the molecular pathways potentially associated with the CC progression. We analyzed 31 LBC cervical samples from women who underwent colposcopy. These samples were divided into two groups: the first group was composed of samples without precursor lesions of CC, considering the control group, referred to as healthy female subjects (HFS; *n* = 11). The second group corresponded to women diagnosed with cervical interepithelial neoplasia grade 3 (CIN 3; *n* = 20). We performed microRNA and gene expression profiling using the nCounter® miRNA Expression Assays (NanoString Technology) and PanCancer Pathways (NanoString Technology), respectively. A microRNA target prediction was performed by mirDIP, and molecular pathway interaction was constructed using Cytoscape. Bidirectional in silico analyses and Pearson's correlation were performed for associated the relation between genes, and miRNAs differentially expressed related cervical cancer progression were performed. We found that the expression of nine microRNAs was significantly higher, two were downregulated (miR-381-3p and miR-4531), and seven miRNAs were upregulated (miR-205-5p, miR-130a-3p, miR-3136-3p, miR-128-2-5p, let-7f-5p, miR-202-3p, and miR-323a-5p) in CIN 3 (fold change ≥ 2 and *p* ≤ 0.05). The miRNA expression patterns were independent of hr-HPV infection. We identified four miRNAs (miR-205-5p, miR-130a-3p, miR-4531, and miR-381-3p) that could be used as biomarkers for CIN 3 in LBC samples through multiple logistic regression analyses. We found 16 genes differentially expressed between CIN 3 and HSF samples (fold change ≥ 2 and *p* ≤ 0.05). We found the correlation between miR-130a-3p and CCND1(*R* = −0.52; *p* = 0.0029), miR-205-5p and EGFR (*R* = 0.53; *p* = 0.0021), and miR-4531 and SMAD2 (*R* = −0.54; *p* = 0.0016). In addition, we demonstrated the most significant pathways of the targets associated with cervical cancer progression (FDR-corrected *p* < 0.001). This study demonstrated that miRNA biomarkers may distinguish healthy cervix and CIN 3 and regulate important molecular pathways of carcinogenesis.

## 1. Introduction

Cervical cancer (CC) corresponds to the fourth most common cancer among women in the world, with 604,127 new cases and 341,831 deaths registered annually [1]. It is estimated that for 20 years from now, there will be an incidence of approximately 798 thousand new cases per year [2]. In developing countries, including Brazil, CC continues to be a significant public health problem [3]. Although the incidence and mortality rates for CC have declined over recent years due to the implementation of prevention programs in developing countries [4, 5], and the 5-year survival rate of patients with advanced CC remains poor [3, 6]. In addition, it is important to note that the rate of overtreatment is still quite high. According to Ebish et al. [7], excessive treatment for high-grade CIN and CC can be defined as the percentage of women being treated without the presence of a high-grade lesion. A meta-analysis [7] carried out to determine overtreatment rates in the management of seeing and treating women referred for colposcopy due to suspected CIN, in order to define the circumstances that support the management of seeing and treating. It was verified that the overtreatment rate in women with a high-grade cervical smear and the low-grade colposcopy impression was 29.3% (95% CI 16.7–41.9%). Therefore, the authors conclude that the pooled overtreatment rate in women with a high-grade smear and high-grade colposcopy impression is elevated, which supports the use of see-and-treat management in this subgroup of women. Thus, discovering biomarkers for cervical lesion progression may reduce the overtreatment of nonprogressive [8] cervical intraepithelial neoplasia grade 2 (CIN 2) and grade 3 (CIN 3).

MicroRNAs (miRNAs) are an abundant class of small noncoding RNA with 20-25 nucleotides in length that modulate the gene expression level by partial base pairing with 3′ untranslated region of their messenger RNAs (mRNAs) [9]. These small molecules regulate the gene expression by direct cleavage of targeted mRNA or inhibiting translation [10], which results in promiscuous interactions: one miRNA often interacts with more than one mRNAs, and one transcript can be targeted by multiple miRNAs [11]. miRNAs are well described as being linked to tumorigenesis, invasion, and metastasis [12]. Several studies have reported the involvement of these molecules from initiation to the progression of CC [8]. Therefore, new miRNA biomarkers for diagnosis, prognosis, and disease prediction are urgently needed [13–17].

In view of this, some studies reported that the differential expression of miRNAs could be used as a biomarker for CC progression [8]. However, these studies are limited to tumor samples from biopsies and surgeries, which are obtained after developing symptoms or when the tumor can be visually detected through routine examination. On the other hand, liquid biopsies are noninvasive systems that enable clinically relevant actions and allow early diagnosis, prognosis, and therapy response [18–21]. The first time the term liquid biopsy was used was that it referred to methods in which a blood sample could be derived from the same diagnostic information from a tissue biopsy sample [22]. In oncology, this term is used in a broad sense to refer to the sampling and analysis of different biological fluids, which are easily accessible [23]. In this context, we used liquid-based cytology (LBC) samples obtained from the cervix scraping to measure the miRNA expression that may be linked to the onset and progression of CC.

We identified four miRNAs (miR-205-5p, miR-130a-3p, miR-4531, and miR-381-3p), differentially expressed between healthy individuals and patients with CIN 3. In addition, we performed in silico analyses and found five molecular pathways likely associated with CC progression, namely, FoxO signaling, microRNAs in cancer, PI3K-Akt signaling, MAPK signaling, and intrinsic apoptosis.

## 2. Materials and Methods

### 2.1. Study Population and Sample Collection

We investigated retrospective cervical cytology samples and clinical data from women who underwent colposcopy in Barretos Cancer Hospital Prevention Department (Brazil) between 2014 and 2015. All cytological samples were collected, as previously described [24]. Briefly, immediately before colposcopy, samples from cervical cell scraping were collected using Cervex-Brush (Rovers Medical Devices, North Brabant, Netherlands). A cervical scraping sample was obtained from each participant and was preserved in the ThinPrep Pap Test (Hologic, Bedford, MA, USA) for further miRNA analyses. According to sample availability, we randomly selected 31 LBC cervical samples using a convenience sampling method: 11 from healthy female subjects (HFS—cervices without precursor lesions of CC, considered as the control group) and 20 from women diagnosed with cervical interepithelial neoplasia grade 3 (CIN 3—the case group). We did not evaluate CIN 1 and 2 because they often show an ambiguous classification [25] and identified the cases using the Prevention Department database. Although our cohort was composed of 31 participants, the model presented a power greater than 0.70 compared to HSF and CIN 3 as variables of interest (Table S1). All samples were stored between 2°C and 8°C.

### 2.2. RNA Isolation

Initially, we washed samples using Dulbecco's Phosphate Buffered Saline (DPBS) to remove blood cells and buffered preservative solution, which could inhibit downstream molecular analyses. Briefly, we used 4 ml LBC preserved in the ThinPrep Pap Test (Hologic, Bedford, MA, USA). These samples were centrifuged for 4 minutes at 1500 g. Then, the supernatant was discarded, and the pellet was resuspended in 500 *μ*l DPBS. This material was centrifuged again under the same conditions. Total RNA extraction was performed with the washed LBC pellet using RecoverAll Total Nucleic Acid Isolation Kit (Thermo Fisher Scientific, Waltham, MA, USA) according to the manufacturer's protocol. After, the purity of total RNA was evaluated by NanoDrop Spectrophotometer v3.7 (Thermo Fisher Scientific, Waltham, MA, USA).

### 2.3. High-Risk HPV Molecular Analysis

High-risk HPV (hr-HPV) molecular analysis was conducted using a Cobas ×480™ device (Roche Molecular Systems, Pleasanton, CA, USA). This platform is an automated amplification using quantitative polymerase chain reactions (PCR) to detect combined results for high-risk (HR) genotypes (HPV-31, 33, 35, 39, 45, 51, 52, 56, 58, 59, 66, and 68) and individual results for HPV-16 and HPV-18, which are the highest risk genotypes. For this analysis, we used aliquots of LCB samples (SurePath™) collected before colposcopy. The assays were performed as indicated by the manufacturer [26].

### 2.4. NanoString nCounter System miRNA and Gene Expression Assay

The expression of the panel containing 800 miRNAs was measured using the nCounter Human v3 miRNA Expression panel with the nCounter Analysis System (NanoString Technologies, Seattle, USA). We prepared 100 ng of total RNA to receive the probes Reporter CodeSet and Capture ProbeSet (nCounter Human v3 miRNA Expression Assay), followed by hybridization. The assay was performed according to the manufacturer's instructions (NanoString Technologies, Seattle, WA, USA) using the NanoString PrepStation. The probe-target complexes were immobilized in the nCounter cartridge, which was placed in the nCounter Digital Analyzer for image capture (280 fields of view). We used the nCounter® PanCancer Pathways panel for gene expression analyses employing the nCounter® Analysis System (NanoString Technologies, Seattle, USA). This panel has 770 genes shown to be implicated in various cancer types and was curated in The Cancer Genome Atlas (TCGA) data. Sample preparation was performed according to the manufacturer's instructions (NanoString Technologies, Seattle, Washington, EUA). Data analysis was performed in Rv3.2.1 (The R Foundation, Viena, Austria) with the NanoStringNorm Package (Bioconductor). We used the quantile method for the normalization of samples in both panels. Normalized data were log2 transformed and used as input for the differential expression analyzes.

### 2.5. In Silico Target Prediction and Pathway Enrichment

We only considered the top 5% of target genes, including genes that had been identified by the Cancer Gene Index data (NCI). Next, to determine the association between miRNA targets and cervical neoplasm involvement, we used the plugin ReactomeFI on Cytoscape (Version 3.6.0, Seattle, WA, USA). We focused on tumor suppressor genes and oncogenes in human cancers according to described in the Catalogue of Somatic Mutations in Cancer (COSMIC) database (https://cancer.sanger.ac.uk/cosmic). Molecular pathway enrichment was performed with a false discovery rate-corrected (FDR-corrected) *p* value lower than 0.001. The interaction network was performed by Cytoscape [27].

### 2.6. Bioinformatics and Statistical Analysis

Student's *t*-test was performed, and a 2-fold change (FC) difference in miRNA expression levels between the groups was evaluated. The ROC curve analysis was performed using the ROCR and PROC package (Bioconductor) in *R*. miRNAs presenting the area under the ROC curve (AUC) ≥ 0.75 were considered as CIN 3-specific miRNA with a good performance. Heatmaps of miRNA expression were generated using the ComplexHeatmap package, considering Euclidean distance. miRNA expression profile was dichotomized into upregulated and downregulated according to the cut-off identified in the ROC curve analysis.

Further, multiple logistic regression was used to identify a panel of miRNAs that may increase the risk for the development of CIN 3. We performed a posthoc calculation to obtain the power given the sample size (*n* = 31), the correlation between the predictor variables (*R*^2^—logistic regression model), the estimated probability for the mean values of the predictor variables (*p*), the level of significance (*α* = 0.05), and the odds ratio estimated by the model (OR). The estimated sample tests' power for the logistic regression model was performed through G^∗^ Power 3.1.9.4 software [28]. The chi-square test was performed to identify associations between the miRNA expression and hr-HPV infection. The correlations between miRNA (miR-205-5p, miR-130a-3p, miR381-3p, and miR-4531) levels and mRNA targets predicted in CC (*CDKN1*, *PTEN*, *PIK3CB*, *STK11*, *CCND1*, *EP300*, *HRAS*, *TGFBR2, PIK3CA*, *EGFR*, *NRAS*, *MAPK1*, *SMAD2*, *SMAD4*, *STAT3*, *MDM2*, *KRAS*, and *ATM*) were analyzed using Pearson correlation analysis. For all statistical analyses, *p* values ≤0.05 were considered significant.

### 2.7. Ethical Approval

This study was approved by the Research Ethics Committee of the Barretos Cancer Hospital under Protocol No. 784/2014 and Certificate for Ethics Assessment (Certificado de Apresentação para Apreciação Ética–CAAE) No. 28174114.1.0000.5437. All information that could be used to identify the study participants was kept confidential and encrypted in the database to ensure the confidentiality of the data and the anonymity of the participants.

## 3. Results and Discussion

### 3.1. Study Population

The clinicopathological features of 31 women (healthy female subjects, HFS, *n* = 11 and CIN 3, *n* = 20) are summarized in Table 1. The mean age of the 31 subjects was 38.4 years. Of the 31 subjects, eight (25.8%) had been positive, and twenty-three (74.2%) had negative hr-HPV test results. From the 20 CIN 3 patients, 95% showed positive hr-HPV test results. We found a higher frequency for all HPV types in the CIN 3 group compared with the control group. In addition, we found that the age of HFS and CIN 3 patients was not a limiting factor for the expression of miRNAs (Table S1).

### 3.2. Identification of miRNA Molecular Signature in High-Grade Precursor Lesions of Cervical Cancer

**Figure 1:**
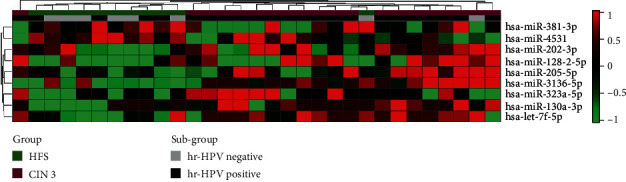
Expression profiles for highly significant miRNAs. We analyzed nCounter miRNA Expression Assay Panel measurements for 800 miRNAs using *t*-tests to discover differences in expression between samples of subjects with CIN 3 (*n* = 20) and those of healthy female subjects (HFS) (*n* = 11). Nine miRNAs displayed statistically significant results (*p* < 0.05, fold change ≥ 2, and AUC ≥ 0.75). Samples are arranged in columns, miRNA expression levels in rows, and both are hierarchically clustered using Euclidean distance with the average linkage of nodes. Red shades indicate increased relative expression; green shades indicate reduced expression; black indicates median expression. The bars above indicate the groups and subgroups used for the analysis. Green indicates the samples from the HFS and the purple subgroup of the CIN 3 group. Black indicates the subgroup hr-HPV positive, and hr-HPV negative is grey.

To identify miRNAs whose expression is dysregulated in CIN 3 compared to HFS, we performed miRNA expression profiling using nCounter Human v3 miRNA Expression panel with 800 miRNA probes. We identified nine miRNAs (miR-205-5p, miR-130a-3p, miR-3136-5p, miR-128-2-5p, let-7f-5p, miR-202-3p, miR-323a-5p, miR-381-3p, and miR-4531) with biomarker potential according to the following criteria: *t*-test (*p* < 0.05), fold change (FC) ≥ 2, and area under the curve (AUC) ≥ 0.75 (Table 2, Figure 1 and S1). From these nine differentially expressed miRNAs, two were downregulated (miR-381-3p and miR-4531), and seven were upregulated (miR-205-5p, miR-130a-3p, miR-3136-3p, miR-128-2-5p, let-7f-5p, miR-202-3p, and miR-323a-5p) in CIN 3. Multiple logistic regression analysis with the nine differentially expressed miRNAs showed that four miRNAs (miR-205-5p, miR-130a-3p, miR-4531, and miR-381-3p) were selected according to their regulation (downregulated and upregulated) (Table 3).

### 3.3. Associations between miRNA Levels and High-Risk HPV Infection

We performed an association analysis between the differential expression of the nine miRNAs and hr-HPV infection, and no significant associations were found. This result allowed us to infer that the expression of the nine miRNAs is independent of hr-HPV infection (Table 4).

### 3.4. Functional In Silico Analysis

We investigated whether the nine miRNAs' main targets differentially expressed between HFS and CIN 3 samples were associated with molecular pathways related to the carcinogenic process. We identified 16 genes predicted as targets (ATM, CCND1, EGFR, EP300, HRAS, KRAS, MAPK1, MCM2, NRAS, PIK3CA, SMAD2, SMAD4, STAT3, STK1, and TGFBR2) of the four miRNAs differentially expressed in this study, miR-130-a-3p, miR-205-5p, miR-381-3p, and miR-4531. 16 genes predicted as targets of the four miRNAs differentially expressed in this study, miR-130-a-3p, miR-205-5p, miR-381-3p, and miR-4531 (FDR-corrected *p* < 0.001). We verified these genes' involvement in critical pathways associated with CC with potential roles of oncogenes and tumor suppressors. In this context, we found that the most significant pathways of the targets associated with cervical neoplasm were FoxO signaling pathway, microRNAs in cancer, PI3K-Akt signaling pathway, MAPK signaling pathways, and intrinsic apoptosis pathways (Table 5).

### 3.5. Gene Expression and Correlation between miRNA and mRNA Target Pairs in CIN 3 from LBC Cervical Samples

**Figure 2:**
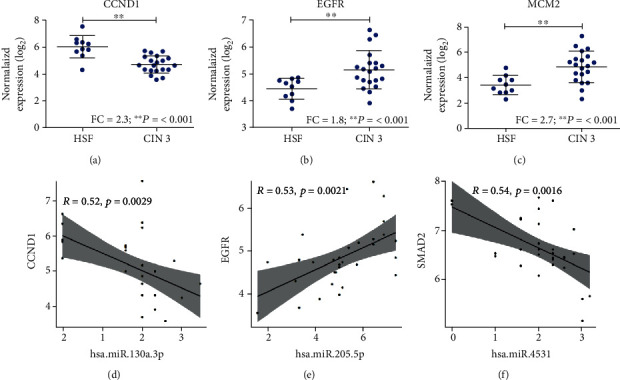
Gene expression and correlation between miRNA and mRNA target pairs in CIN 3 patients. (a) The expression of *CCND1* in CIN 3 and HFS samples. (b) The expression of *EGFR* in CIN 3 and HFS samples. (c) The expression of *MCM2* in CIN 3 and HFS samples. (d) Pearson's correlation analysis between has-miR-130a-3p and *CCND1* (Pearson's correlation coefficient, *R* = −0.52; *p* = 0.0029). (e) Pearson's correlation analysis between has-miR-205-5p and *EGFR* (Pearson's correlation coefficient, *R* = 0.53; *p* = 0.0021). (f) Pearson's correlation analysis between has-miR-4531 and *SMAD2* (Pearson's correlation coefficient, *R* = −0.54; *p* = 0.0016). HFS: healthy female subjects (*n* =11); CIN 3: cervical intraepithelial neoplasia grade 3 (*n* = 20). ^∗∗^*p* value <0.01. Student's *t*-test was performed to evaluate the gene expression levels between the biological groups.

Next, we analyzed the expression of 16 genes previously predicted by in silico analyses in cases with HSF and CIN 3 and validated the differential expression of these 16 genes between the CIN 3 and HFS samples using the panel PanCancer Pathways (NanoString Technologies) (Figure S2). In CIN 3, expression levels of *CCND1* (FC = −2.3; *p* ≤ 0.001; Figure 2(a)) were significantly higher than in HFS samples. On the other hand, *EGFR* (FC = 1.8; *p* ≤ 0.001; Figure 2(b)) and *MCM2* (FC = 2.7; *p* ≤ 0.001; Figure 2(c)) expression levels were significantly decrease in control samples.

Moreover, to gain further insight into the miRNA-target RNA dynamics, we integrated data from previously collected mRNA measurements with miRNA expression findings. Three significant correlations were observed in CIN 3 samples. We found that the has-miR-130a-3p expression was negatively correlated with the *CCND1* expression in the CIN 3 group (*R* = −0.52; *p* = 0.0029; Figure 2(d)). In addition, has-miR-205-5p was positively correlated with *EGFR* in the CIN 3 group (*R* = 0.53; *p* = 0.0021; Figure 2(e)). Importantly, *SMAD2* (*R* = −0.54; *p* = 0.0016; Figure 2(f)) mRNA levels increased with declining miR-4531 levels.

## 4. Discussion

This is the first study to demonstrate the nanostring-derived miRNA expression profile as a biomarker for CIN 3 detection. However, other studies have already described the miRNA expression in high-grade lesions, CIN 2 and 3, using other methodological approaches [25, 29–31]. Lu et al. 2019 [29] evaluated the potential functions of miRNAs in HPV 16 replication and determined the detailed mechanism for regulating the IFN immune response using microarray and qRT-PCR assays. On the other hand, Snoek et al. 2019 [25] showed that the deregulated miRNA expression associated with CIN 3 and CC development could be detected by sRNA-Seq in HPV-positive self-samples. Previous miRNA microarray analysis revealed that miR-218 is downregulated in CC tissues [31]. Subsequently, another study validated the miR-218 expression using qRT-PCR, and the authors verified that this miRNA was downregulated in the tumor tissues and plasma of patients with CC. This finding suggests that miR-218 may have antitumor functions in CC [30]. We used new technology to identify the differential miRNA expression compared to previously describe molecular triage markers in CIN and CC.

In this sense, our analysis revealed good clinical performance of a miRNA molecular signature (miR-205-5p, miR-130a-3p, miR-3136-5p, miR-128-2-5p, let-7f-5p, miR-202-3p, miR-323a-5p, miR-381-3p, and miR-4531), derived from linear regression, with high discriminatory power for CIN 3 detection using LBC samples since these are minimally invasive samples and can, therefore, be considered as liquid biopsy. Our analyses revealed that the differential expression profile of these nine miRNAs occurs regardless of the hr-HPV infection status. Moreover, we observed that patients from the HFS group are older than those from the CIN 3 group. However, this factor was not a limiting effect for the expression of miRNAs since we did not evidence statistically significant differences between the expression of miRNAs by age groups (<39 years old and ≥39 years old) (Table S1). Furthermore, we identified four miRNAs (miR-205-5p, miR-130a-3p, miR-4531, and miR-381-3p) after multiple logistic regression analyses. We suggest that these miRNAs can be used as biomarkers for CIN 3 detection in LBC samples.

miR-205-5p and miR-130a-3p markers were overexpressed in CIN 3 samples compared to HFS samples, while miR-4531 and miR-381-3p were downregulated in CIN 3. Evidence has shown that deregulation of miR-205-5p increases according to the precursor lesions stage and is also involved in key processes for tumor development and maintenance, such as the increase of cell proliferation and invasion [32, 33].

We identified nine differentially expressed miRNAs when comparing CIN 3 tumors to normal samples. Among the nine miRNAs, miR-130a-3p was overexpressed; this miRNA is a potential oncomiR since it regulates cell proliferation, migration, and apoptosis inhibition in CC tissue and cell lines [34]. In this context, Zhang et al. [34] also showed that the overexpression of miR-130a-3p increased the sensitivity to cisplatin and 5-fluorouracil in five cell lines (KYSE-70, KYSE-140, KYSE-180, KYSE-270, KYSE-410, and KYSE-520). To confirm these tumor-promoting activities, the authors found that this miRNA inhibition resulted in cell cycle arrest.

miR-130a-3p also regulates Bcl-2, which activates Bax and caspases-9/3, leading to cell apoptosis via p53 [35]. On the other hand, some studies have also demonstrated the potent role of miR-381-3p as a progression tumor biomarker, such as in breast cancer, in which its overexpression reduced cell proliferation [36]. In contrast, Shang et al. (2018) found that miR-381-3p was downregulated in CC cell lines compared with the normal tissue [37]. Regarding miR-453 and miR-381-3p, we identified downregulation in CIN 3 samples compared to HFS.

Indeed, we observed that few studies evidence the differential expression of miRNAs with CC progression, especially using LBC samples. There is no consensus among studies measuring the expression of miR-205-5p in samples of preneoplastic lesions and cancer. We also verified that data related to miR-130a-3p and miR-381-3p corroborated with what was found in the literature.

We found several common target genes involved in CC carcinogenesis based on the nine differentially expressed miRNAs. These genes have already been described as being associated with uterine cervix neoplasms. The major signaling pathways they regulate are FoxO signaling, microRNAs in cancer, PI3K-Akt signaling, MAPK signaling, and intrinsic apoptosis pathways. According to these results, one can speculate that these miRNAs can regulate targets of the pathways listed above and act CC progression. However, functional studies involving these miRNAs are needed to clarify the understanding the role of these molecules in CC.

This work provides important data on new miRNAs as potential noninvasive biomarkers that can be used to differentiate the progression of CC among women with CIN 3 and HFS. However, some limitations should be considered. Prospective studies with larger cohorts are required to define the differential expression of these miRNAs. Finally, we encourage future studies to demonstrate the functional role in vitro and in vivo of these identified miRNAs to determine if they affect the progression of CC.

## 5. Conclusions

In conclusion, we identified nine miRNAs (miR-205-5p, miR-130a-3p, miR-3136-5p, miR-128-2-5p, let-7f-5p, miR-202-3p, miR-323a-5p, miR-381-3p, and miR-4531) that were able to distinguish HFS and CIN 3 groups. Furthermore, we showed that these nine miRNAs are not linked to hr-HPV infection, indicating that these miRNAs' differential expression may be a factor independent of hr-HPV infection. In addition, using multiple logistic regression analyses, we identified four miRNAs (miR-205-5p, miR-130a-3p, miR-4531, and miR-381-3p) that can be used as biomarkers for CIN 3 in LBC samples. From our in silico functional assay, we suggest that these miRNAs may be involved with the progression of CC.

## Figures and Tables

**Table 1:** Clinical features of the investigated patients.

	Features	HFS (*n* = 11)	CIN 3 (*n* = 20)	*p* value^∗^
Age, years	Median (min-max)	42 (32-69)	35 (19-48)	0.036

		Value, *n* (%)	Value, *n* (%)	

Categorized age	<30 years	−	6 (30.0%)	
	30–49 years	8 (72.7%)	14 (70.0%)	
	>50 years	3 (27.3%)	−	

hr-HPV	Negative	7 (63.7%)	1 (5.0%)	
	HPV 16	−	5 (25.0%)	
	HPV 18	−	1 (5.0%)	
	HPV others	4 (36.3%)	8 (40.0%)	
	HPV 16 + others	−	5 (25.0%)	

HFS: healthy female subjects without cervical intraepithelial neoplasia (CIN); CIN 3: cervical intraepithelial neoplasia grade 3; hr-HPV: high-risk human papillomavirus; Min: minimum; Max: maximum. ^∗^The Mann–Whitney test was performed to evaluate age between the biological groups. *p* < 0.05.

**Table 2:** Sensitivity, specificity, AUC, fold change, and *p* value of differentially expressed miRNAs between CIN 3 and HFS samples.

miRNA	Sensitivity	Specificity	AUC	Fold change	*p* value^∗^
miR-381-3p	64%	70%	0.77	-2.1607	0.0043
miR-4531	73%	70%	0.77	-2.1607	0.0047
miR-205-5p	70%	91%	0.78	3.6693	0.0100
miR-130a-3p	75%	55%	0.78	3.0042	0.0078
miR-3136-5p	90%	73%	0.77	2.0138	0.0057
miR-128-2-5p	70%	73%	0.77	2.4596	0.0048
let-7f-5p	80%	82%	0.77	2.0138	0.0320
miR-202-3p	65%	82%	0.77	3.0042	0.0078
miR-323a-5p	45%	100%	0.75	2.0138	0.0089

^∗^Significant (*p* < 0.05) student's t-test results from the comparison of the miRNA expression levels between the biological groups. AUC: area under the curve. *p* < 0.05.

**Table 3:** Multiple logistic regression analysis of miRNAs differentially expressed (upregulated and downregulated) in CIN 3 samples compared with HFS.

	miRNA	OR	CI	*p* value
Upregulated	miR-130a-3p	4.286	1.305-14.081	0.016
	miR205-5p	2.094	0.989-4.432	0.050

Downregulated	miR-4531	0.030	0.001-0.684	0.028
	miR-381-3p	0.024	0.001-0.660	0.027

**Table 4:** Associations between nine miRNAs differentially expressed and hr-HPV infection (FC ≤ 1.5, *p* < 0.05, and AUC ≥ 0.75).

miRNAs		hr-HPV		*p* value^∗^
	Regulation	Negative	Positive	
		*n* (%)	*n* (%)	
miR-205-5p	Upregulated	7 (38.9%)	11 (61.1%)	0.095
	Downregulated	1 (7.7%)	12 (92.3%)	

miR-130a-3p	Upregulated	7 (30.4%)	16 (69.6%)	0.642
	Downregulated	1 (12.5%)	7 (87.5%)	

miR-3136-5p	Upregulated	4 (30.8%)	9 (69.2%)	0.689
	Downregulated	4 (22.2%)	14 (77.8%)	

miR-128-2-5p	Upregulated	6 (35.3%)	11 (64.7%)	0.240
	Downregulated	2 (14.3%)	12 (8.7%)	

Let-7f-5p	Upregulated	7 (33.3%)	14 (66.7%)	0.222
	Downregulated	1 (10.0%)	9 (90.0%)	

miR-202-3p	Upregulated	7 (31.8%)	15 (68.2%)	0.379
	Downregulated	1 (11.1%)	8 (88.9%)	

miR-323a-5p	Upregulated	8 (30.8%)	18 (69.2%)	0.291
	Downregulated	−	5 (100.0%)	

miR-381-3p	Upregulated	5 (20.8%)	19 (79.2%)	0.335
	Downregulated	3 (42.9%)	4 (57.1%)	

miR-4531	Upregulated	5 (21.7%)	18 (78.3%)	0.393
	Downregulated	3 (37.5%)	5 (62.5%)	

^∗^Chi-square test was used to identify associations between miRNA expression and hr-HPV infection.

**Table 5:** Top 5 pathways related to the best target candidates of the miRNAs differentially expressed between HFS and CIN 3.

Pathway	Genes	*p* value^∗^
FoxO signaling pathway	CDKN1A, CDKN1B, PTEN, PIK3CB, STK11, CCND1, EP300, HRAS, TGFBR2, PIK3CA, EGFR, NRAS, MAPK1, SMAD2, SMAD4, STAT3, MDM2, KRAS, ATM	*p* < 0.001
MicroRNAs in cancer	CDKN1A, CDKN1B, PTEN, BRCA1, CCND1, MYC, EP300, HRAS, TP63, PIK3CA, CCNE1, MET, TP53, NOTCH2, NOTCH1, EGFR, NRAS, SOCS1, ABL1, MAPK1, STAT3, APC, BCL2, MDM2, KRAS, ATM	*p* < 0.001
PI3K-Akt signaling pathway	CDKN1A, CDKN1B, FLT4, PTEN, PIK3CB, BRCA1, CCND3, STK11, CCND1, MYC, MYB, HRAS, HSP90AA1, PIK3CA, CCNE1, MET, TP53, EGFR, NRAS, MAPK1, IL2, CDK4, BCL2, MDM2, KRAS	*p* < 0.001
MAPK signaling pathways	RB1, CDKN1A, CDKN1B, CCND3, CCND1, MYC, CHEK2, EP300, CCNE1, TP53, ABL1, SMAD2, SMAD4, CDK4, MDM2, ATM	*p* < 0.001
Intrinsic apoptosis pathways	PIK3CB, HRAS, PIK3CA, DDIT3, TP53, NRAS, MAPK1, JUN, BCL2, FAS, BAX, KRAS, ATM	*p* < 0.001

^∗^False discovery rate-corrected (FDR-corrected) *p* value lower than 0.05 was used to identify the molecular cervical cancer pathways.

## Data Availability

The datasets analyzed in the current study are available from the corresponding author on reasonable request.

## Supplementary Materials

Supplementary Materials Table S1: associations between nine miRNAs differentially expressed and categorized age (<39 and ≥39 years old) in both biological groups (HSF and CIN 3). Table S2: calculation of power and effect size for the realization of the logistic regression model. Figure S1: expression levels of the miRNAs signature in LBC cervical. Normalized gene expression levels (log2) obtained from miRNA expression assay (NanoString Technologies, Seattle, Washington, EUA) data of 9 miRNAs in LBC cervical (11 controls (green), 20 high-grade CIN (red)). ^∗^*p* < 0.05, ^∗∗^*p* < 0.01. Student's t-test was performed to evaluate the miRNA expression levels between the biological groups. Figure S2: gene expression levels in LBC cervical. Normalized gene expression levels (log2) obtained from nCounter PanCancer Pathways Panel (NanoString Technologies, Seattle, Washington, EUA) data of 16 genes previously predicted by in silico analyses in LBC cervical (11 controls = HSF “Healthy Subject Female”), 20 CIN 3 (cervical intraepithelial neoplasia grade 3)). ^∗∗^*p* < 0.01. Student's *t*-test was performed to evaluate the gene expression levels between the biological groups.
