# Supplementary material for: MicroRNA Biomarkers of High-Grade Cervical Intraepithelial Neoplasia in Liquid Biopsy
Source: Biomed Res Int. 2021 Apr 13;2021:6650966. doi: 10.1155/2021/6650966 (PMC8060087; doi:10.1155/2021/6650966)
Supplement: Supplementary Materials — Table S1: associations between nine miRNAs differentially expressed and categorized age (<39 and ≥39 years old) in both biological groups (HSF and CIN 3). Table S2: calculation of power and effect size for the realization of the logistic regression model. Figure S1: expression levels of the miRNAs signature in LBC cervical. Normalized gene expression levels (log2) obtained from miRNA expression assay (NanoString Technologies, Seattle, Washington, EUA) data of 9 miRNAs in LBC cervical (11 controls (green), 20 high-grade CIN (red)). ∗p < 0.05, ∗∗p < 0.01. Student's t-test was performed to evaluate the miRNA expression levels between the biological groups. Figure S2: gene expression levels in LBC cervical. Normalized gene expression levels (log2) obtained from nCounter PanCancer Pathways Panel (NanoString Technologies, Seattle, Washington, EUA) data of 16 genes previously predicted by in silico analyses in LBC cervical (11 controls = HSF “Healthy Subject Female”), 20 CIN 3 (cervical intraepithelial neoplasia grade 3)). ∗∗p < 0.01. Student's t-test was performed to evaluate the gene expression levels between the biological groups. [file 6650966.f1.docx]

**Table S1.** Associations between nine miRNAs differentially expressed and categorized age (< 39 and ≥ 39 years old) in both biological groups (HSF and CIN 3).

| **miRNA(median)** | **HFS** | | **CIN 3** | | **p-value*** |
| --- | --- | --- | --- | --- | --- |
|  | **< 39 years** | **≥ 39 years** | **< 39 years** | **≥ 39 years** |  |
| **hsa-miR-205-5p** | 4.929 | 4.700 | 5.226 | 6.642 | 0.647 |
| **hsa-miR-130a-3p** | 0.000 | 2.000 | 2.000 | 2.161 | 0.133 |
| **hsa-miR-381-3p** | 2.293 | 2.000 | 1.585 | 1.000 | 0.788 |
| **hsa-miR-4531** | 2.454 | 2.322 | 2.000 | 1.793 | 0.601 |
| **hsa-miR-3136-5p** | 1.793 | 1.585 | 2.322 | 2.696 | 0.200 |
| **hsa-miR-128-2-5p** | 0.000 | 0.000 | 1.293 | 1.793 | 0.619 |
| **hsa-let7f-5p** | 0.793 | 1.585 | 2.000 | 2.000 | 0.261 |
| **hsa-miR-202-3p** | 1.293 | 1.000 | 2.000 | 2.000 | 0.522 |
| **hsa-miR-323a-5p** | 1.000 | 0.000 | 1.293 | 0.500 | 0.217 |

*Chi-Square test was used to identify associations between miRNA expression and categorized age in both biological groups. HFS: Healthy female subjects - without cervical intraepithelial neoplasia (CIN). CIN 3: cervical intraepithelial neoplasia grade 3.

**Table S*2*.** Calculation of power and effect size for the realization of the logistic regression model.

| **Regulation** | **miRNA** | **R^2^** | **p** | **OR** | **Power** |
| --- | --- | --- | --- | --- | --- |
| Upregulated | miR-130a-3p | 0.053 | 0.733 | 4.286 | 0.729 |
|  | miR-205-5p |  |  | 2.094 | 0.323 |
| ­­­­­Downregulated | miR-4531 | 0.070 | 0.841 | 0.030 | 0.979 |
|  | miR- 381 |  |  | 0.024 | 0.983 |

R2: squared coefficient of multiple correlation with other covariates. OR: odds ratio associated in covatiate of interest. For this calculation, the G* Power 3.1.9.4 software was used.


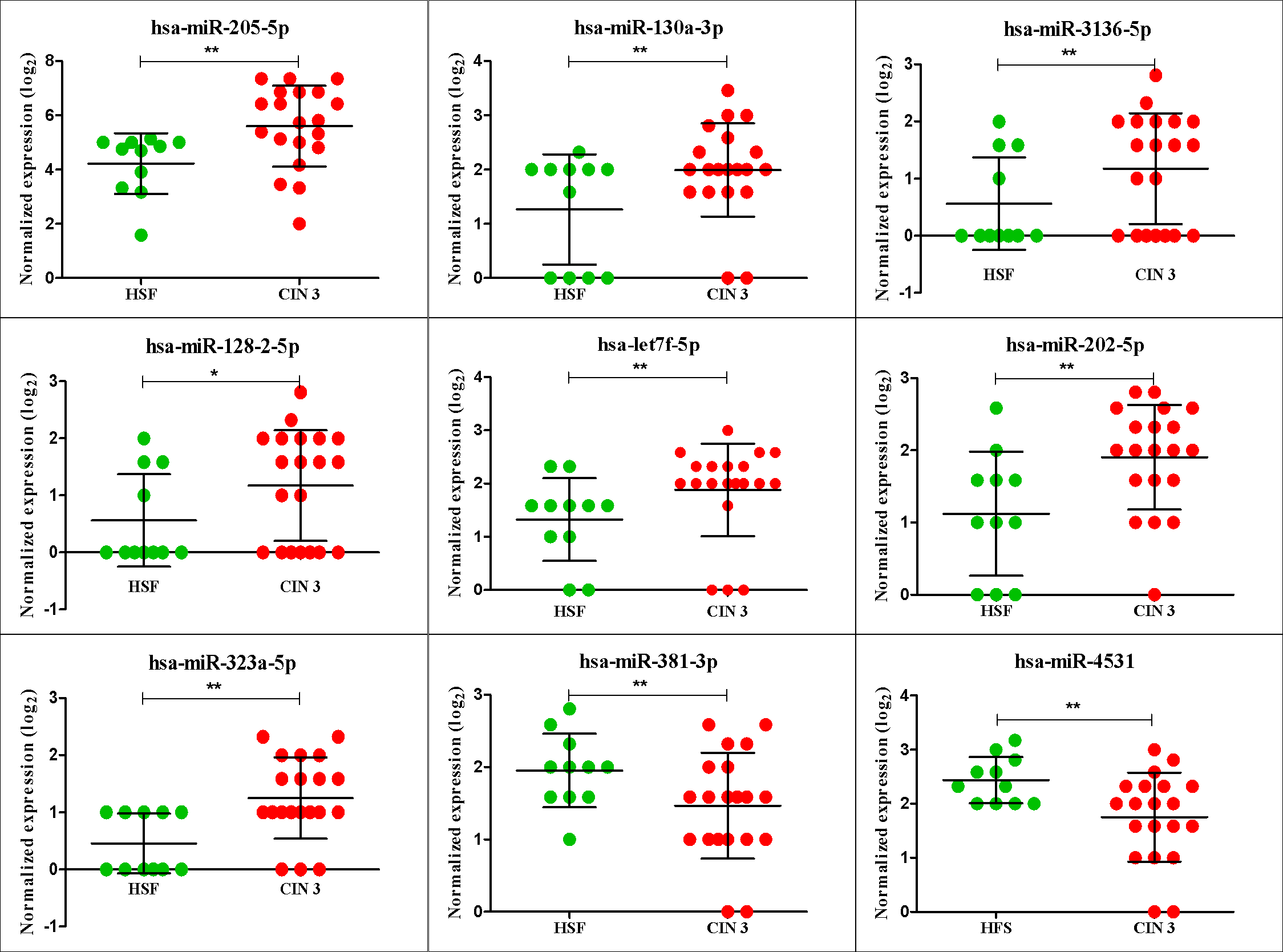


**Figure S1:** Expression levels of the miRNAs signature in LBC cervical. Normalized gene expression levels (log_2_) obtained from miRNA expression assay (NanoString Technologies, Seattle, Washington, EUA) data of 9 miRNAs in LBC cervical (11 controls (green), 20 high-grade CIN (red)). *p<0.05, **p<0.01. Student's t-test was performed to evaluate the miRNA expression levels between the biological groups.


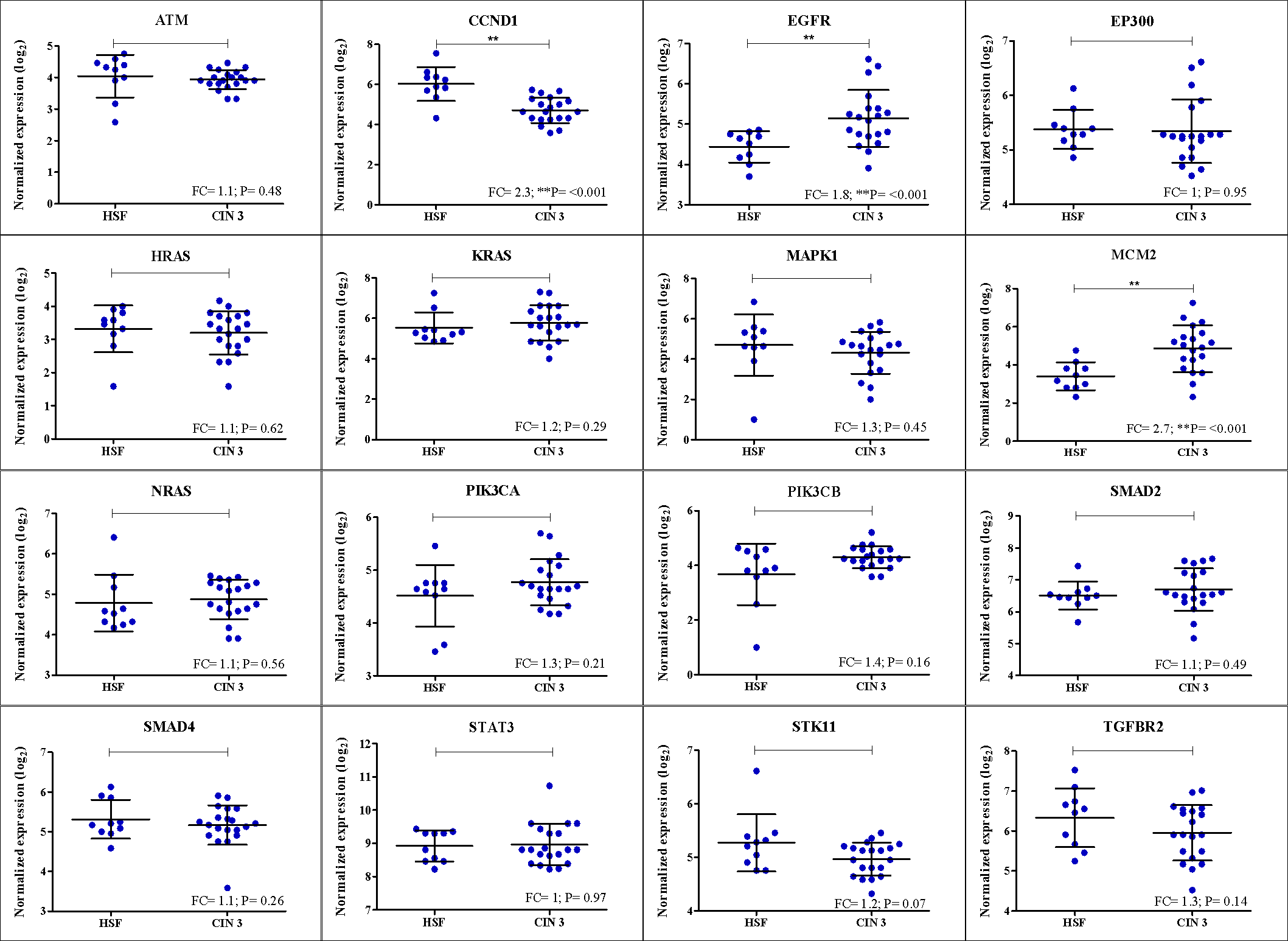


**Figure S2:** Gene expression levels in LBC cervical. Normalized gene expression levels (log_2_) obtained from nCounter PanCancer Pathways Panel (NanoString Technologies, Seattle, Washington, EUA) data of 16 genes previously predicted by in silico analyses in LBC cervical (11 controls = HSF ‘Healthy Subject Female’), 20 CIN 3 (Cervical Intraepithelial Neoplasia grade 3)). **p<0.01. The student's t-test was performed to evaluate the gene expression levels between the biological groups.
